# Transcriptomic Properties of HER2+ Ductal Carcinoma In Situ of the Breast Associate with Absence of Immune Cells

**DOI:** 10.3390/biology10080768

**Published:** 2021-08-12

**Authors:** Marie Colombe Agahozo, Marcel Smid, Ronald van Marion, Dora Hammerl, Thierry P. P. van den Bosch, Mieke A. M. Timmermans, Chayenne J. Heijerman, Pieter J. Westenend, Reno Debets, John W. M. Martens, Carolien H. M. van Deurzen

**Affiliations:** 1 Department of Pathology, Erasmus MC Cancer Institute, P.O. Box 2040, 3000 CA Rotterdam, The Netherlands; m.agahozo@erasmusmc.nl (M.C.A.); r.vanmarion@erasmusmc.nl (R.v.M.); t.vandenbosch@erasmusmc.nl (T.P.P.v.d.B.); 2 Department of Medical Oncology, Erasmus MC Cancer Institute, P.O. Box 2040, 3000 CA Rotterdam, The Netherlands; m.smid@erasmusmc.nl (M.S.); d.hammerl@erasmusmc.nl (D.H.); a.timmermans@erasmusmc.nl (M.A.M.T.); c.heijerman@erasmusmc.nl (C.J.H.); j.debets@erasmusmc.nl (R.D.); j.martens@erasmusmc.nl (J.W.M.M.); 3 Laboratory for Pathology Dordrecht, 3318 AL Dordrecht, The Netherlands; pwestenend@paldordrecht.nl

**Keywords:** breast ductal carcinoma in situ, HER2 amplification, transcriptome assay, next generation sequencing, RNA, protein, immunohistochemistry, TIL density

## Abstract

**Simple Summary:**

Tumor-infiltrating lymphocytes (TILs) are likely to play a role in the biological behavior of HER2+ ductal carcinoma in situ (DCIS). To prevent invasiveness, the potential of targeted immune-modulating treatment of HER2+ DCIS has been explored. We identified a 29-gene expression profile that was associated with the density of TILs. These genes included CCND3, DUSP10 and RAP1GAP, which may guide towards more rationalized choices with respect to immune-mediated therapy in HER2+ DCIS, such as targeted vaccine therapy.

**Abstract:**

The identification of transcriptomic alterations of HER2+ ductal carcinoma in situ (DCIS) that are associated with the density of tumor-infiltrating lymphocytes (TILs) could contribute to optimizing choices regarding the potential benefit of immune therapy. We compared the gene expression profile of TIL-poor HER2+ DCIS to that of TIL-rich HER2+ DCIS. Tumor cells from 11 TIL-rich and 12 TIL-poor DCIS cases were micro-dissected for RNA isolation. The Ion AmpliSeq Transcriptome Human Gene Expression Kit was used for RNA sequencing. After normalization, a Mann–Whitney rank sum test was used to analyze differentially expressed genes between TIL-poor and TIL-rich HER2+ DCIS. Whole tissue sections were immunostained for validation of protein expression. We identified a 29-gene expression profile that differentiated TIL-rich from TIL-poor HER2+ DCIS. These genes included *CCND3*, *DUSP10* and *RAP1GAP*, which were previously described in breast cancer and cancer immunity and were more highly expressed in TIL-rich DCIS. Using immunohistochemistry, we found lower protein expression in TIL-rich DCIS. This suggests regulation of protein expression at the posttranslational level. We identified a gene expression profile of HER2+ DCIS cells that was associated with the density of TILs. This classifier may guide towards more rationalized choices regarding immune-mediated therapy in HER2+ DCIS, such as targeted vaccine therapy.

## 1. Introduction

Ductal carcinoma in situ (DCIS) of the breast is characterized by a proliferation of neoplastic cells confined within the duct [1,2]. When left untreated, DCIS can progress into invasive breast cancer (IBC) [3,4]. Several markers for progression have been proposed, including high nuclear grade, large DCIS size and the overexpression of human epidermal growth factor receptor 2 (HER2) [4,5,6,7,8]. HER2+ DCIS accounts for 23–37% of all DCIS cases and is associated with high nuclear grade, large diameter and comedonecrosis [9,10]. HER2 overexpression in DCIS has also been reported as a biomarker for local recurrence and upstaging to IBC in the final excision specimen after a biopsy diagnosis [10,11,12,13]. Since HER2+ IBC has an aggressive biological behavior, optimal early treatment is needed to minimize the risk of DCIS progression to IBC. Previous studies reported the potential of targeted treatment of HER2-enriched DCIS, including vaccine therapy, to prevent invasive disease [14,15,16,17,18].

The first HER2 vaccine study in HER2+ DCIS was conducted over a decade ago, using dendritic cells pulsed with HER2 peptides [14]. This study included 13 patients, who were treated with this vaccine after a biopsy diagnosis of HER2+ DCIS. After surgical resection, partial pathologic response was reported in seven patients and complete tumor regression in one patient [14]. Other studies demonstrated the feasibility and safety of the vaccine, whereby complete tumor regression was seen in up to 30% of the patients [15,16,17,18]. Although these results are promising, identifying patients that benefit from the vaccine remains challenging. The vaccine gives rise to an increased number of anti-HER2 CD4+ T cells in peripheral blood, but this does not always result in a local immune response [17]. Lowenfeld et al. demonstrated that all DCIS patients with pathologic complete response had elevated levels of anti-HER2 CD4+Th1 cells in their sentinel lymph nodes [17]. This emphasizes the importance of a loco-regional immune response. However, the pre-vaccination local immune response could also be important with respect to the efficacy of immune-modulating therapies.

HER2+ breast cancer is associated with a pronounced local immune response, marked by large numbers of tumor-infiltrating lymphocytes (TILs), both in DCIS as well as IBC lesions [19,20]. With regard to IBC, numerous studies reported an association between large numbers of TILs and improved prognosis in triple negative and HER2+ cases [21,22,23,24]. Additionally, patients with large numbers of TILs seem to have a better response to immune-modulating therapies such as immune checkpoint inhibitors [25]. With respect to the prognostic role of DCIS-associated TILs, data remain limited. In general, large numbers of TILs have been associated with invasive recurrence. This could be related to the distribution of TILs across DCIS subtypes, since TILs are mainly seen in high grade, HER2+ or triple negative cases [19,26,27,28]. The prognostic role of DCIS-associated TILs within DCIS subtypes (i.e., HER2+ DCIS with large numbers of TILs versus HER2+ DCIS with small numbers of TILs) is not well known, since the majority of recent studies did not correct for DCIS subtype. Nonetheless, HER2+ DCIS presents with a large number of CD8+ T cells, compared to HER2−DCIS [29,30,31].

HER2 overexpression in DCIS is a potential immune antigen. This might contribute to the relatively high frequency of TILs observed in HER2+ DCIS compared to HER2+ IBC [19,32,33,34,35,36,37]. However, not all HER2+ DCIS cases present with increased numbers of TILs. This suggests that other molecular alterations of the tumor cells could also contribute to the density of TILs. Several methods to manipulate the tumor in order to convert TIL-poor tumors in TIL-rich tumors have been reported in breast cancer [38,39,40]. The identification of (potentially targetable) transcriptomic alterations of tumor cells that are associated with the density of TILs could guide further development of immune-mediated therapy in HER2+ DCIS, such as targeted vaccine therapy. The aim of this study was therefore to compare the gene expression profile of TIL-poor HER2+ DCIS to that of TIL-rich HER2+ DCIS.

## 2. Patients and Methods

### 2.1. Study Cohort and Histopathological Assessment

We included treatment-naïve patients diagnosed with DCIS (without an invasive component) at the Erasmus Medical Center in Rotterdam or the Laboratory for Pathology in Dordrecht, between 2004 and 2016. Patients for this study were selected from a previously described cohort of pure DCIS cases. All included patients were estrogen receptor (ER) and progesterone receptor (PR) negative and HER2 positive (ER-PR-HER2+). ER (SP1; Ventana) and PR (1E2; Ventana) were previously scored according to Dutch guidelines for IBC and HER2 (4B5; Ventana) was scored according to international guidelines [41,42]. Additionally, Ki67 (30-9; Ventana) and P53 (Bp53011; Ventana) were determined by automated immunohistochemistry using the Ventana Benchmark ULTRA (Ventana Medical System Inc., Oro Valley, AZ, USA), Ki67 was scored as the percentage of positive cells and P53 was scored as wild type, absent or overexpressed. The detailed staining procedure is listed in Supplementary Table S2.

Using all diagnostic hematoxylin and eosin-stained whole sections of excision specimens, the TIL density of these HER2+ DCIS cases was semi-quantitatively scored as previously described by Agahozo et al. [31].

Using these scores, cases with minimal/mild TIL density and matched cases with severe TIL density were included. Cases with 0–30% of the DCIS-associated stroma occupied by TILs were classified as TIL poor and cases with >50% of the DCIS-associated stroma occupied by TILs were classified as TIL rich. These cases were matched based on age, histologic grade, presence of comedonecrosis and tumor diameter. Patients with IBC within the first 6 months after diagnosis were excluded. According to the code of conduct of the Federation of Medical Scientific Societies in the Netherlands, there was no need for informed consent, since only encoded leftover patient material was used for this study [43]. This work was approved by the Medical Ethics Committee (MEC 02.953).

### 2.2. Micro-Dissection and RNA Isolation

**Figure 1:**
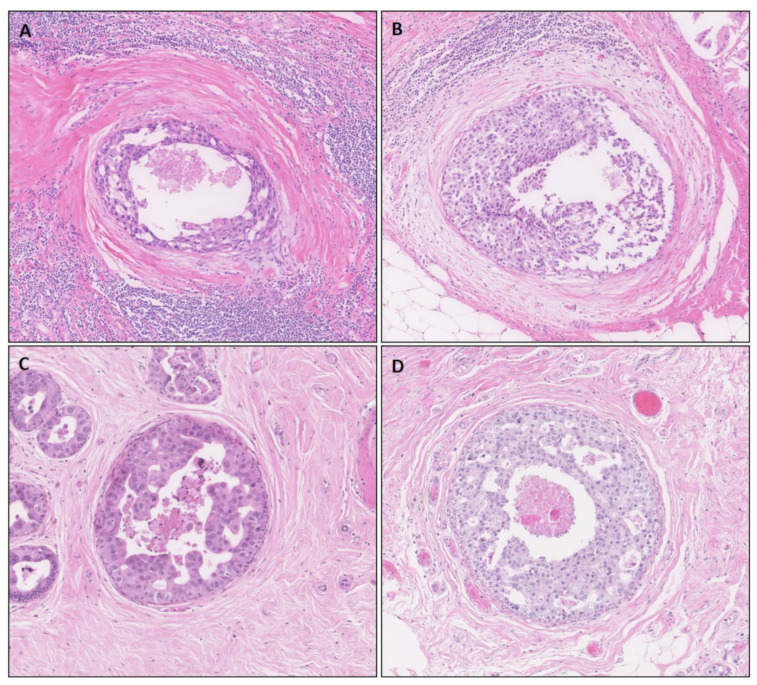
Histological representation of TIL-rich (**A**,**B**) DCIS. Neoplastic cells are surrounded by a TIL-poor area of reactive stroma and, subsequently, a zone of many TILs. (**C**,**D**) TIL-poor DCIS cases.

Tumor cells from both TIL-rich and TIL-poor cases were micro-dissected (Figure 1). DCIS cells were micro-dissected separately from the TILs, to ensure pure tumor cell samples. Prior to micro-dissection, the areas for micro-dissection were marked using the last 4 µm thick hematoxylin and eosin-stained slide of 11 sequential slides. The remaining 10 slides with 10µm thick sections of formalin-fixed paraffin-embedded tissues of DCIS were dewaxed and rehydrated, followed by hematoxylin staining. Following hematoxylin staining, DCIS cells were micro-dissected manually using a sterile scalpel under a stereomicroscope (Zeiss, Oberkochen, Germany) and stored in RNAse/DNase-free tubes containing RNALater (Thermo Fisher, Waltham, MA, USA). Samples were stored at −80 °C until further RNA isolation.

Prior to isolation, samples were spun down, RNALater was removed and samples were washed with ethanol. Next, RNA was isolated using the AllPrep RNA/DNA formalin-fixed paraffin-embedded isolation kit (Qiagen, Hilden, Germany) according to the manufacturer’s instructions. After isolation, RNA concentrations were measured by NanoDrop (Thermo Fisher Scientific) and PicoGreen by Qubit (Thermo Fisher Scientific). Additionally, we performed a quality control on all RNA samples using RT-qPCR (Bioline) to validate the amplification and MultiNA (Shimadzu) to quantify the fragment size and concentration, as previously described by Siewerts et al. [44]. Samples were then stored at −80 °C.

### 2.3. Targeted RNA Sequencing

The Ion AmpliSeq Transcriptome Human Gene Expression Kit was used for targeted RNA sequencing. Using 5 to 43 ng of formalin-fixed paraffin-embedded RNA, cDNA was generated using the SuperScript^®^ VILO™ cDNA Synthesis Kit, followed by target region amplification using the Ion AmpliSeq Transcriptome Human Gene Expression core panel. After partial digestion of the primers, adapters were ligated to amplicons and purified. The generated library was quantified by qPCR with the Ion Library TaqMan Quantitation kit. Pooled libraries, with 6 to 8 samples per pool, were templated on the Ion Chef and sequenced using a 540 chip on the Ion GeneStudio S5 Prime system. Finally, transcription data were generated as raw read counts using the ampliSeqRNA (target region: hg19_AmpliSeq_Transcriptome_21K_v1) plugin.

### 2.4. Data Processing and Analysis

R v3.6 was used for data analysis. First, raw read counts were normalized using EdgeR [45]. After normalization, a principal component analysis (PCA) showed large differences in overall expression distribution according to the batch of the samples. To correct for this, genes were removed if not expressed in all samples from a single batch. The remaining missing data were imputed per gene by using the median expression level of the gene. These data (8753 genes) were used for input in ComBat to correct for the batch effects [46]. Finally, a PCA was used to confirm correction of the batch effect. The PCA after batch correction did not separate the samples based on TIL-rich or TIL-poor cases.

In order to measure the purity of our tumor RNA samples, we assessed the potential admixture of TILs using a previously described TIL signature [47]. This TIL signature was generated using breast cancer samples with a high and low TIL count, combined with gene expression data from these samples (GEO54219). This analysis resulted in a 152-probe signature that highly correlated with the percentage of TILs in the specimens. Recently, this signature was validated in an independent set and associated with subtype-specific prognosis in breast cancer, which included 109 genes [48]. A Mann–Whitney rank sum test was used to analyze differentially expressed genes between TIL-poor and TIL-rich HER2+ DCIS. We used genes with a *p*-value of <0.05 to generate an s-curve of the fold change to determine a cut-off. The top and bottom tail of the s-curve included genes with a fold change of >4 or <−4. This included the genes from which we selected the ones suitable for immunohistochemistry. Next, we searched for the protein function in breast cancer and immune regulation via the PubMed database and Uniprot.org. We selected three genes for immunohistochemistry based on their association with breast cancer, immune regulation and, finally, antibody availability.

### 2.5. Immunohistochemistry

From these 29 differentially expressed genes, three genes, CCND3, DUSP10 and RAP1GAP, were selected for further analysis of protein expression. CCND3 and DUSP10 were previously associated with survival in IBC and RAP1GAP was associated with breast cancer invasiveness [49,50,51,52,53]. Finally, these genes were also described in relation to immune regulation [51,53,54].

To determine protein expression, we stained 4 µm thick formalin-fixed paraffin-embedded whole tissue sections by automated immunohistochemistry using the Ventana Benchmark ULTRA (Ventana Medical System Inc.). We also included breast cancer cell lines in triplicate, with known microarray gene expression data [55]. Detailed staining procedures are listed in Supplementary Table S1. Briefly, following deparaffinization and heat-induced antigen retrieval, tissue samples were incubated with DUSP10 (polyclonal; Abcam), RAP1GAP (Y134; Abcam) or CCND3 (DCS2.2; Abcam) (Supplementary Table S1). After incubation, hematoxylin II counter stain was incubated for 8 min and followed by a blue coloring reagent for 8 min according to the manufacturer’s instructions (Ventana). The protein expression of DUSP10 and RAP1GAP was semi-quantitively scored according to the H-score, whereby the staining intensity (0–3) was multiplied by the total percentage of positive epithelial cells (0–100) [54]. CCND3 was scored as the percentage of positive epithelial cells. IBC cell lines were scored independently by two observers. The average score was used for analyses.

### 2.6. Statistical Analysis

All clinical and protein data were statistically analyzed using SPSS Statistics 21 (IMB). A chi-square test was used to test for associations between the TIL status and categorical variables. For continuous variables, a Mann–Whitney U test was used to test for differences in case they were not normally distributed. A Student’s *t*-test was used to compare means of continuous variables. Correlations were tested using Spearman’s rho. Results were considered significant with a *p*-value < 0.05.

## 3. Results

### 3.1. Clinicopathological Patient Characteristics

A total of 23 patients were included, of which there were 11 TIL-rich and 12 TIL-poor cases, with a median age at diagnosis of 56, ranging from 37.0 to 73.0 years. General patient and DCIS characteristics are depicted in Table 1. The majority of DCIS cases were high grade, with comedonecrosis and calcification. The median Ki67 expression was 14.2% and the majority of the patients (69.5%) had mutated p53 protein expression, whereby overexpression was detected in 47.8% of the cases. These clinicopathological characteristics were similar between TIL-rich and TIL-poor patients (Supplementary Table S2).

### 3.2. Differentially Expressed Genes on RNA Level

In order to validate the purity of our micro-dissected tumor cells, we determined the TIL score of each sample using normalized (log2 scale), imputed and batch corrected data, using the TIL signature (109 genes) [48]. Then, we compared the TIL score from TIL-rich DCIS samples to that of TIL-poor samples. There was no difference in the mean TIL score between TIL-rich and TIL-poor samples (*p* = 0.294, TIL score = 2.1 vs. 2.5, respectively).

Besides analyzing the TIL signature as a whole, we also investigated individual genes of the signature, but we did not identify any TIL signature gene that was significantly differentially expressed between the micro-dissected tumor cells of TIL-rich and TIL-poor samples. Thus, these data are suitable to identify differentially expressed genes between TIL-rich and TIL-poor samples that are indeed derived from transcriptomic differences of the tumor cells and not related to the potential admixture of TILs.

**Figure 2:**
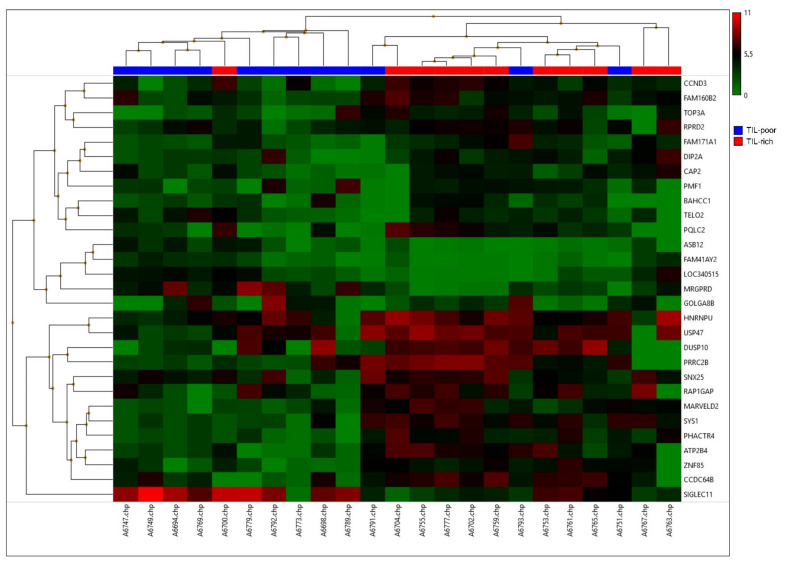
A hierarchical cluster of the top 29 differentially expressed genes. High expression is depicted in red and low expression is depicted in green. TIL-poor DCIS is depicted in blue and TIL-rich DCIS is depicted in red.

After data processing and analysis, 29 differentially expressed genes were selected (Table 2). Many of these genes (14 out of 29) were involved in the cell cycle or protein transportation. A hierarchical clustering was performed on these genes (Figure 2). This hierarchical clustering demonstrates two clear groups, whereby TIL-rich DCIS cases are clustered together and TIL-poor DCIS cases are clustered together, with an exception of three samples. Two samples were TIL poor (A6751 and A6793) and clustered with TIL-rich samples and one was a TIL-rich sample (A6700) that clustered with TIL-poor samples.

### 3.3. Differentially Expressed Genes on Protein Level

The mRNA expression levels of CCND3, DUSP10 and RAP1GAP were higher in TIL-rich DCIS compared to TIL-poor DCIS, with a median Log2 value of 5.60 vs. 3.50 for CCND3, 7.17 vs. 4.44 for DUSP10 and 6.20 vs. 3.58 for RAP1GAP, respectively. To validate this differential expression on the protein level, immunohistochemical analysis was performed.

**Figure 3:**
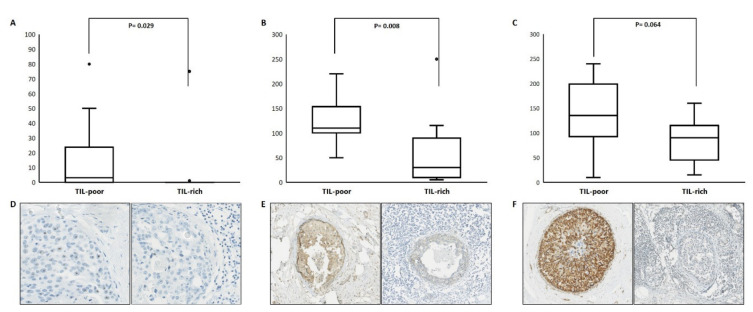
Protein expression of CCND3, DUSP10 and RAP1GAP according to TIL-poor and TIL-rich DCIS. The upper panels depict the protein expression level as % of CCND3 (**A**), H-scores of DUSP10 (**B**) and RAP1GAP (**C**). The lower panels depict representative immunohistochemical images of CCND3 (**D**) at 40×, DUSP10 (**E**) and RAP1GAP (**F**) at 10× magnification.

Protein expression of CCND3, DUSP10 and RAP1GAP is depicted in Figure 3, including representative images (Figure 3D–F). We scored the nuclear CCND3 protein expression as the percentage of positive DCIS cells. CCND3 protein expression was detected in 10 out of 23 patients of whom the majority (*n* = 8) were TIL poor. TIL-rich DCIS showed a lower CCND3 protein expression compared to TIL-poor DCIS, *p* = 0.029, with a median percentage of positive cells of 0.0% vs. 3.0%. After dichotomization, using the presence or absence of CCDN3 expression (cut-off at 1%), a chi-square test showed a *p*-value of 0.036. The presence of CCND3 protein expression was associated with TIL-poor DCIS. Cytoplasmic DUSP10 and RAP1GAP protein expression was detected in all patients, which were scored with an H-score. The level of DUSP10 protein expression was lower in TIL-rich DCIS compared to TIL-poor DCIS, *p* = 0.008, with a median H-score of 30.0 vs. 110.0, respectively. We also observed lower RAP1GAP protein expression in TIL-rich DCIS compared to TIL-poor DCIS, with a median H-score of 90.0 vs. 135.0, respectively. However, with a *p*-value of 0.064, this difference did not reach significance.

Next, RNA and protein expression of CCND3, DUSP10 and RAP1GAP was evaluated on breast cancer cell lines with known gene expression data to assess the correlation between gene and protein expression of these genes. In total, 52 breast cancer cell lines were included. There was no correlation between the mRNA expression and protein expression. Spearman’s rho values were −0.150, 0.073 and 0.198 for CCND3, DUSP10 and RAP1GAP, respectively (*p* = 0.292, 0.613 and 0.164, respectively). This was also the case for ER-HER2+ cell lines (n = 11).

## 4. Discussion

HER2+ IBC is associated with increased numbers of TILs, which are associated with better prognosis. With regard to DCIS, data regarding the clinical relevance of TILs remain limited, but previous data suggested a potential role of DCIS-associated TILs with respect to biological behavior [19,27,31,56]. Additionally, not all HER2+ DCIS cases present with increased numbers of TILs. Differently expressed genes of HER2+ DCIS cells might play a role in the density of TILs and could therefore contribute to DCIS progression. In this study, we compared the gene expression profile of TIL-poor HER2+ DCIS to that of TIL-rich HER2+ DCIS.

Clinicopathological characteristics did not differ between TIL-poor and TIL-rich DCIS cases. This included Ki67 and P53 protein expression, which are generally linked to larger number of TILs in IBC [56,57,58]. Since DCIS cells and DCIS-associated TILs are anatomically separated in the majority of cases, as illustrated in Figure 1, manual micro-dissection under a stereomicroscope has no substantial effect on the purity of tumor cells. Based on the gene expression profile of micro-dissected DCIS cells, there was indeed no indication that the purity of tumor cells in our samples could have affected the results. Overall, we identified 29 differentially expressed genes potentially playing a role in the density of TILS in HER2+ DCIS, of which many were involved in the cell cycle or protein transportation.

Out of these 29 differentially expressed genes, we analyzed three genes of interest at the protein level. These genes are CCND3, DUSP10 and RAP1GAP and are involved in cell proliferation, differentiation and migration. Additionally, they were previously described in breast cancer and cancer immunity. CCND3 belongs to the cyclin D family and functions as a regulator of the CDK kinases, which are involved in the differentiation and proliferation of tumor cells [59]. It is predominantly amplified in basal-like breast cancer and is associated with breast cancer progression and reduced overall and disease-free survival [49,59,60,61]. In our study, TIL-rich DCIS cases were associated with higher CCND3 gene expression compared to TIL-poor DCIS. On the contrary, protein expression of CCND3 was predominantly found in TIL-poor DCIS. The discordance between mRNA and protein expression might indicate posttranslational regulation of CCND3 expression. Indeed, ubiquitin-mediated degradation of CCND3 by FBXL2 has been demonstrated in lung cancer, while in breast cancer cells, the RNA-binding protein IMP-3 can regulate CCND3 protein expression by directly binding to its mRNAs [62,63]. Further corroborating our finding is the fact that the presence of CDK4/6, which are regulated by CCNDs, has previously been associated with the absence of TILs in IBC [64]. Additionally, inhibiting these kinases increased T cell infiltration and activation of effector T cells in (murine) tumors [65,66]. We therefore suggest a potential involvement of CCND3 expression in the regulation of TILs. However, regulation could also occur the other way around. Nonetheless, the actual causal role of TILs in this regulation has yet to be investigated.

DUSP10 is a phosphatase that is upregulated in HER2+ IBC and reduces the inflammatory response [67,68,69]. Similar to CCND3, we found higher DUSP10 gene expression levels in TIL-rich DCIS compared to TIL-poor cases, while the opposite was found at the protein level. Our data suggest that DUSP10 protein expression, despite having a lower transcript level, might be linked to suppressing TIL density in HER2+ DCIS. This is also in line with data demonstrating that reduced DUSP10 expression in airway epithelial cells potentiated the release of CXCL8 and CXCL1 and increased IL-1β levels, which promote immune infiltration [70].

RAP1GAP is a GTPase-deactivating protein, which controls the activity of Rap1. It has been reported as a tumor suppressor gene in various solid tumors, including IBC [53,71]. Specifically, downregulation of RAP1GAP has been demonstrated to occur at the switch from DCIS to IBC [53]. Its expression is increased in the DCIS stage and drops in the IBC stage. However, this study did not find the presence of TILs. We demonstrated increased RAP1GAP gene expression in TIL-rich HER2+ DCIS compared to TIL-poor DCIS, but observed the opposite regarding the protein expression. While these results might seem contradictory, RAP1GAP is regulated at the posttranslational level. The RAP1GAP protein can be degraded though the PLK1-mediated ubiquitination pathway [71]. In turn, PLK1 expression is positively associated with an increased number of TILs in IBC [72,73,74]. This suggests that TIL-rich HER2+ DCIS could have increased PLK1 expression, which may lead to more RAP1GAP degradation on the protein level. However, the mechanism of how RAP1GAP and PLK1 expression is linked to TILs remains unknown.

## 5. Conclusions

In summary, we identified 29 genes in HER2+ DCIS that are associated with the density of TILs, which supports a potential role of these genes in the local immune response and, consequently, the biological behavior of DCIS. Considering our small cohort and lack of significant FDR *p*-values, these genes need to be further validated and evaluated. However, our data support that the density of TILs in HER2+ DCIS is a consequence of mechanisms whereby genetic modifications might need to be altered at the protein level. The identified gene classifier may guide towards more rationalized choices with respect to immune-mediated therapy in HER2+ DCIS, such as targeted vaccine therapy.

## Figures and Tables

**Table 1:** Patient and DCIS characteristics.

Characteristic	n/Median	%/Range
Age	56	37.0–73.0
DCIS size (cm)	3.8	0.9–9.0
Grade		
Low	0	0
Intermediate	1	4.3
High	22	95.7
Comedonecrosis		
Absent	3	13
Present	20	87
Growth Pattern		
Solid	15	65.2
Cribriform	7	30.4
Papillary	1	4.3
p53		
Wild type	7	30.4
Aberrant	11	47.8
Null	5	21.7
Ki67 (%)	14.2	3.0–25.0

**Table 2:** Differentially expressed genes properties.

Gene	*p*-Value	Fold Change (High–Low)	Role in (Breast) Cancer	Reference	General Function (Uniprot.org, accessed on 11 July 2021)
HNRNPU	0.02	4.07	Upregulated in pancreatic ductal adenocarcinoma, mediates invasion and migration in pancreatic ductal adenocarcinoma cell lines	Shen et al., Med Sci Monit 2018, Sutaria et al., Noncoding RNA 2017	DNA/RNA-binding protein, **cell cycle/transcription**
MRGPRD	0.01	−5.09	Unkown	n.a.	**Transcription**
RPRD2	0.04	4.05	Mutated in Burkitt lymphoma	Kaymaz et al., Mol Cancer Res. 2017	Regulatory protein, **transcription**
TOP3A	0.02	4.89	Unknown	n.a.	DNA/RNA-binding protein, **cell cycle/transcription**
ZNF85	0.02	4.12	Unknown, overexperssed in SCLC cell lines	Loiselle et al. Heliyon 2016	DNA/RNA binding protein, **transcription**
BAHCC1	0.01	4.47	Predicts survival in melanoma, upregulated in hepatocellular carcinoma	Gao et al., Biomed Res Int 2020, Nalesnik et al., Am J Pathol 2012	Chromatin-binding protein, **cell cycle**
CCND3	0.02	4.29	Amplified in basal-like breast cancer, correlates with reduced overall suvival in breast cancer, discriminates inflammatory breast cancer from non-inflammatory breast cancer	Smid et al., Nat Comm 2016, Ding et al., Cancer Medicine 2019, Keyomarsi et al., N Engl J Med 2002, Lerebours et al., BMC 2008	Regulatory protein, **cell cycle**
PHACTR4	0.02	5.61	Suggested tumorsupressor in various cancers including breast cancer, overexpression inhibits cell proliferation and invasion in hepatocellular carcinoma by inhibiting IL6/Stat3 pathway	Solimini et al., PNAS 2012, Cao et al., Eur Rev Med Pharmacol Sci. 2016	Regulatory protein, **cell cycle**
PMF1	0.04	5.65	Regulates the expression of SSAT in breast cancer cell lines, methilation associated with bladder cancer progression	Husbeck et al., Biochem Biophys Res Commun 2003, Aleman et al., Clin Cancer Res. 2008	Involved in **cell cycle**
TELO2	0.05	4.52	Associated with oncogenic profile in breast cancer cell line	Morais-Rodrigues et al., Gene 2020	**Cell cycle**
USP47	0.03	4.33	Promotes EMT (mortality and disasociation) in breast cancer cells	Silvestrini et al., J Proteomics 2020	Ubiquitin-specific protease, negative regulator of **cell cycle**
DUSP10	0.01	6.62	Mediates immune response, increased DUSP10 downregulates inflammation and overexpressed in HER2+ breast cancer, high expression associated with reduced relapse-free survival in ER+ wt P53 breast cancer	Jiménez-Martínez et al., Int J Mol Sci. 2019, Hrstka et al., Mol Onc 2015	Enzyme, **proliferation and differentiation**
PRRC2B	0.02	11.98	Unknown, somatic variant found in T-cell lymphoma	Donner et al., Fam Cancer 2019	RNA binding, **cell differentiation**
RAP1GAP	0.03	6.18	Tumorsupressive in several cancers, inhibits progression in endometrial cancer, increased in ductal carcinoma in situ compared to invasive breast cancer, reduced expression enhances invasion	Tamate et al., Biochem Biophys Res Commun. 2017, Shah et al., Neoplasia 2018	Regulatory protein, **differentiation and proliferation**
ASB12	0.05	−4.28	Unknown	n.a.	E3 ubiquitin protein, **translation**
DIP2A	0.05	4.68	Promotes FSTL1 immune resistance and correlates with poor prognosis in non-small cell lung cancer patients	Kudo-Saito et al., Cell Rep 2018	Regulatory protein, **developmental protein**
FAM171A1	0.00	5.16	Increased expression in invasive vs. in situ breast carcinoma, correlates with loss of ER and formation of mammospheres (in cell lines) (suggested to increase metastatic potential) in triple negative breast cancer, suggested prognostic marker in triple negative breast cancer (cell lines), ref.	Rsila et al., The American Joural of Pathology 2019, Sanawar et al., Ongegenesis 2019, Bao et al., Cell Death Dis. 2019	**Cell shape/mortality**
LOC340515	0.04	−4.02	Unkown	n.a.	
MARVELD2	0.02	5.49	Generally described in pancreas and liver carcinoma. Overexpression (tricellulin) associated with unfavorable pronnosis in primary liver carcinomas, decreased expression correlates with poor prognosis in pancreatic adenocarcionma	Somoracz et al., Pathol Oncol Res. 2014	**Cell–cell junction**
SIGLEC11	0.03	−8.59	Unknown	n.a.	Regulatory protein
ATP2B4	0.01	8.89	Metastasis surpressor of BRAF mutated melanoma cells, overexpression plays a role in chronic lymphocytic leukemia pathogenesis, lower ATP2B4 mRNA expression in invasive breast cancer tissue samples compared to normal breast tissue	Hegudus et al., Int J Cancer 2017, Johnston et al., Mol Cell Proteomics 2018, Varga et al., BMC Cancer 2028	Enzyme/catalyzes calcium **transport**
CCDC64B	0.01	4.15	Unknown	n.a.	Rab GTPase binding, **transport**
GOLGA8B	0.04	−4.27	Associated with shorter overall survival in patients with renal cell carcinoma, associated with tumor progression and prognosis in prostate cancer	Wang et al., Journal of Cellular Biochemistry 2018, Cheng et al., J Cell Mol Med. 2020	**Protein transport**
PQLC2	0.00	4.13	Overexpression promotes cell growth and tumor formation of gastric cancer in nude mice. Suppression/inhibition causes cell death of cancer cells and suppressed growth	Jeung et al., Cancer Sci 2019	**Protein transport**
SNX25	0.01	4.44	Unknown	n.a.	**Protein transport**
SYS1	0.04	8.32	Overexpressed in cervical cancer	Wu et al., Mol Med Rep 2018	**Protein transport**
CAP2	0.02	4.70	Associated with PR expression and decreased overall survival in breast cancer, suggested prognostic marker in gastric cancer	Xu et al., Oncol Rep 2016, Li et al., Pathol Oncol Res. 2020	Regulatory protein, **unknown**
FAM160B2	0.03	4.26	Enhances tumorigenesis in hepatocellular carcinoma (RAI16)	Wang et al., Carcinogenesis 2012	Unknown
FAM41AY2	0.02	−4.38	Unknown	n.a.	Unknown

n.a. = not applicable, **bold** = overall function. Transcription, Cell cycle, Differentiation, Others, Transport, Unknown.

## Supplementary Materials

The following are available online at https://www.mdpi.com/article/10.3390/biology10080768/s1, Supplementary Tables S1 and S2.
