# Supplementary material for: Transcriptomic Properties of HER2+ Ductal Carcinoma In Situ of the Breast Associate with Absence of Immune Cells
Source: Biology (Basel). 2021 Aug 12;10(8):768. doi: 10.3390/biology10080768 (PMC8389698; doi:10.3390/biology10080768)
Supplement: Supplementary file 1 [file biology-10-00768-s001.zip › biology-1087636-supplementary.pdf]

Supplementary tables

Supplementary Table 1: Antibody characteristics and used protocol for whole tissue IHC

Antibody	Type	Concentration /dilution	Company	Clone	Lot number	Procedure	Incubation time
**ER**	Anti-Rabbit	1 µg/ml	Ventana	SP1	F02583	Ultraview CC1 64'	32 minutes
**PR**	Anti-Rabbit	1 µg/ml	Ventana	1E2	Y08684	Ultraview CC1 36'	12 minutes
**HER2neu**	Anti-Rabbit	6 µg/ml	Ventana	4B5	E06192	Ultraview CC1 36'	32 minutes
**Ki67**	Anti-Rabbit	2 µg/ml	Ventana	30-9	F01342	Ultraview CC1 36'	28 minutes
**P53**	Anti-Mouse	2.5 µg/ml	Ventana	Bp53-11	F27098	Ultraview CC1 64'	4 minutes
**CCND3**	Anti-Mouse	1:100	Abcam	DCS2.2	GR3223822-11	Optiview CC1 32'	120 minutes
**DUSP10**	Anti-Rabbit	1:100	Abcam	polyclonal	GR3213653-10	Ultraview CC1 36'	32 minutes
**RAP1GAP**	Anti-Rabbit	1:3000	Abcam	Y134	GR257921-12	Ultraview CC1 36'	32 minutes

Supplementary table 2: Patient and DCIS characteristics according to TIL density

	TIL-poor (n=12)	TIL-rich (n=11)	p-value
Age (in years; mean - range)	57.3 (38.0-72.0)	54.5 (37.0-73.0)	0.689
DCIS size (in cm; mean - range)	3.2 (0.9-6.50)	4.38 (1.5-9.0)	0.341
Grade (%)			
Low	0 (n.a.)	0 (n.a.)	1
Intermediate	1 (100)	0 (0)	
High	11 (50)	11 (50)	
Comedonecrosis (%)			
Absent	2 (67)	1 (33)	1
Present	10 (50)	10 (50)	
Growth Pattern (%)			
Solid	9 (60)	6 (40)	0.427
Cribriform	3 (43)	4 (57)	
Papillary	0 (0)	1 (100)	
p53 expression (%)			
Wild type	4 (57)	3 (43)	0.822
Abarrent	6 (55)	5 (45)	
NULL	2 (40)	3 (60)	
Ki67 (mean - range)	14.8 (3.0-25.0)	13.5 (5.0-25.0)	0.507

n.a. = not appplicable
